# Reflect, Inspire, Strengthen, and Empower 2.0 Program: Advancing Careers and Leadership for Women Physician Staff in an Academic Institution

**DOI:** 10.1089/whr.2023.0094

**Published:** 2024-02-01

**Authors:** Ji Yun Kang, Ivana T. Croghan, Caroline L. Matchett, Laura E. Raffals, Anne A. Schletty, Tammy R. Monson, Karen M. Fischer, Erin M. Pagel, Karthik Ghosh, Anjali Bhagra

**Affiliations:** ^1^ Department of Human Resources, Mayo Clinic, Rochester, Minnesota, USA.; ^2^ Division of General Internal Medicine, Mayo Clinic, Rochester, Minnesota, USA.; ^3^ Department of Internal Medicine, Mayo Clinic School of Graduate Medical Education, Mayo Clinic College of Medicine and Science, Rochester, Minnesota, USA.; ^4^ Division of Gastroenterology and Hepatology, Mayo Clinic, Rochester, Minnesota, USA.; ^5^ Internal Medicine Administrative Services, and Mayo Clinic, Rochester, Minnesota, USA.; ^6^ Division of Clinical Trials and Biostatistics, Mayo Clinic, Rochester, Minnesota, USA.

**Keywords:** appreciative coaching, career development, emotional intelligence, leadership self-efficacy, physician leadership, well-being

## Abstract

**Background:**

To study the effects of the Reflect, Inspire, Strengthen, and Empower (RISE) 2.0 Program designed for professional development of women staff. Topics included emotional intelligence, appreciative coaching, resilience, and strategic career development.

**Methods:**

The RISE 2.0 program was held between September 2020 and February 2021. After each session, program satisfaction surveys were sent to evaluate whether session objectives were met. Professional network, professional mentor, and professional goals were surveyed at the introductory session and at 1 month after the program ended. Survey data about leadership self-efficacy, motivation to lead, and well-being were collected at the introductory session (baseline) and at months 1 and 3 to evaluate the sustainability of program outcomes.

**Results:**

Of the 71 notified, 41 (58%) committed to the program. Results increased for having a robust professional network from baseline to month 1 for very good (7.3% to 13.3%) and excellent (19.5% to 40%). Those who responded favorably to setting and attaining ambitious goals increased from 78.1% to 93.3%. For leadership self-efficacy, all except 2 respondents reported an increase in ratings from baseline to month 3. Motivation to lead changed only slightly. Well-being scores fluctuated as affected by daily needs and fulfillment. For 10 of 15 respondents, well-being increased overall from baseline to month 1 or 3, from month 1 to 3.

**Conclusions:**

Based on participant evaluations and feedback, the RISE 2.0 program received positive responses overall in achieving its learning goals. The program exhibited promise in fostering career advancement and leadership development, particularly when assessed using indicators predictive of successful leadership, such as self-efficacy, motivation to lead, and overall wellbeing.

## Introduction

Today's health care environment is more complex than ever, characterized by fast-changing technologies, cultural shifts, and policy adjustments. Leaders are challenged to maintain core operations, yet, deal with a highly ambiguous future. In addition, an unprecedented talent crisis exists in health care, causing organizations to upskill existing employees while actively recruiting and working to retain high-performing medical professionals. These trends demand highly skilled and effective leaders. Leaders need to be resilient, adaptable, and committed to developing and empowering others to lead through transformational changes. Leaders need to be powerful influencers who inspire and empower staff to think creatively and be willing to change.

Despite the demands on health care leaders, targeted and customized leadership development efforts have not kept up with the demand. As a result, many health care organizations are faced with weak bench strength or physicians not stepping up to take informal or formal leadership roles. Effective leaders influence not only staff well-being, performance, and satisfaction but also patient outcomes.

Recognizing the need to invest in future leaders, a career development and leadership program was created called Reflect, Inspire, Strengthen, and Empower (RISE) 2.0 to broaden the meaning of leadership. This program follows an innovative pilot program that was launched in October 2019.^1^ The program comprises professional engagement, satisfaction, and fulfillment. The purpose of this study was to examine the effect of the RISE 2.0 leadership program for women faculty and advanced practice practitioners on participants' leadership self-efficacy, motivation to lead, and well-being. All of these outcome variables have been shown to influence overall leadership effectiveness^2,3^ and are reported to be interrelated.^4,5^

### RISE 2.0 program structure

**FIG. 1.**
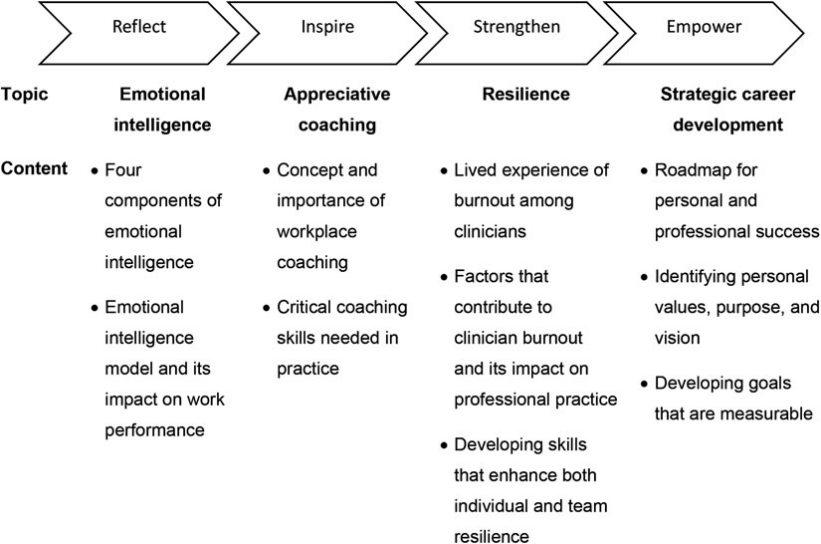
The RISE 2.0 Program Content. RISE, Reflect, Inspire, Strengthen, and Empower.

The RISE 2.0 program that comprised four components (Fig. 1) was designed for professional development focusing on leadership, staff engagement, and joy and well-being at work for women faculty within the Division of General Internal Medicine and Division of Gastroenterology and Hepatology. The program took place between September 2020 and February 2021. Each session was held during the noon hour and via Zoom to accommodate the need for physical distancing during the pandemic. Facilitators were internal and external experts in each field. The participants engaged in follow-up action items between sessions, such as readings and peer coaching. Our study was designed to allow us to evaluate overall satisfaction and reception of this program by women physicians engaged in a busy clinical practice, to assess outcomes using participant satisfaction ratings, and to measure leadership self-efficacy, motivation to lead, and well-being. Ultimately, our goal was to provide insight for developing larger formal programs.

### Theories and concepts

Some leadership theories and concepts that helped build the RISE 2.0 program and are important for understanding the research study follow.

#### Leadership

We used a definition of leadership for this program from Northouse^6^: “Leadership is a process whereby an individual influences a group of individuals to achieve a common goal.” This definition emphasizes leadership as a process and not as a trait or a characteristic. It also emphasizes social interaction and is not confined by any specific role or title. Anyone with leadership influence can be considered as a leader of a group regardless of formal title or role.

#### Women physician leadership

Women physicians in academic medicine have experienced gender disparities in their career advancement for multiple reasons: the burden of family responsibilities, an implicit bias against women making them feel less valued or disempowered, generally less familiarity with organizational resources, insufficient mentoring, fewer networking opportunities, and lack of recognition for accomplishments.^7–9^ The definition of leaders as a social influence has changed with the impact of societal changes and with changes in organizational thoughts on the capabilities of an effective leader, both of which have moved away from previously held views of mainly masculine leadership qualities, such as charisma, command, and control. The prominence of a newer people and relationship-oriented leadership no longer requires women to conform to former qualities of leadership or to gender norms.

Understanding the values and challenges of women physicians and supporting them with needed resources and development opportunities will help them achieve career development, work satisfaction, and well-being. The RISE 2.0 program took into consideration the needs of women physicians in promoting relationship building, peer coaching, collective reflection, inspiration, and empowerment.

#### Transformational leadership

Transformational leadership is a process that changes and transforms people^6,10^ and includes assessing an individual's motives, satisfying their needs, and invoking an exceptional form of influence. Transformational leaders engage with others and create connections that raise others' levels of motivation and morality.^11^ Bass and Riggio^12^ argue that transformational leaders raise staff consciousness about the importance and values of shared visions and goals. Idealized influence, inspirational motivation, intellectual stimulation, and individualized consideration are four components of this transformational leadership. Leaders transform and motivate others by (1) helping them become more aware of the importance of goals, (2) helping them transcend their own interests for the sake of the team and organization, and (3) activating their higher order needs.^13^ Common characteristics of transformational leadership include^10^

- Articulating a clear and appealing vision
- Providing a clear plan for how the vision can be attained
- Demonstrating confidence and optimism
- Expressing confidence in others
- Demonstrating action to emphasize key values
- Leading by example

Theories of transformational leadership strongly support emotional processes (having others feel valued, empowered, recognized, and motivated) as being as important if not more important than rational processes.

#### Emotional intelligence

Today's health care workplace is fraught with emotions. People are fulfilled when goals are achieved but are anxious about complexity and uncertainty, stressed and overwhelmed with workloads, threatened with new technology, sad for not being seen or acknowledged, yet, optimistic about the future and what can be accomplished. Emotions have important roles in the workplace because emotions contain important data about people. When people express emotion, others can better understand how they are interpreting a certain situation or how they are responding to it. Emotions influence leader effectiveness by helping people develop collective goals, instill an appreciation of the importance of work, and generate and maintain enthusiasm, motivation, confidence, optimism, cooperation, and trust.^13^ Emotionally intelligent leaders leverage emotion for influencing, decision-making, and creative problem solving. Emotionally intelligent leaders are known to improve job satisfaction and team performance.^14,15^

Emotional intelligence is also reported to affect psychological well-being.^16,17^ Lin et al.^18^ studied emotional intelligence and its relationship with well-being in a group of 73 surgical residents by using the Dupuy Psychological General Well-Being Index, the Maslach Burnout Inventory, and the Beck Depression Inventory. They found that emotional intelligence was a significant predictor of mental and physical health.

Various studies have provided theoretical relationships between emotional intelligence and transformational leadership.^12,18–20^ A branch of emotional intelligence called perceiving emotion encompasses the ability of persons to identify emotions in themselves and others, a trait important to inspiring and motivating with considerations for individuals. Using emotion is another branch of emotional intelligence that allows persons to generate emotion to facilitate judgment.^19^ Leaders who generate positive emotions facilitate creativity in themselves, allowing for compelling visions that can challenge others to think beyond their usual comfort level.^14^

#### Appreciative coaching

Cox et al.^21^ described coaching as “a human development process that involves structured, focused interaction and the use of appropriate strategies, tools, and techniques to promote desirable and sustainable change for the benefit of the coachee and potentially other stakeholders.”^22^ Coaching is based on a partnership between the coach and coachee, with the goal to maximize the coachee's potential. This partnership makes coaching a powerful leadership skill that can be used for any developmental conversations, including career advancement, skills development (e.g., communication), delegation, decision-making, and stress or time management.

Appreciative coaching has its foundation in positive psychology and is derived from appreciative inquiry, a concept originally formed by Cooperrider et al.^23^ as a method for effecting organizational change.^24^ Rather than focusing on what is not working, the Cooperrider's method focuses on recognizing and understanding what causes persons to do and be their best. In appreciative inquiry, meaning and reality are created through appreciative human communications. The coaching process includes asking coachees open-ended questions to engage them in self-reflection and to have them listen for values, assumptions, emotion, and purpose while acknowledging affirmation, empowerment, and inspiration.

## Methods

Institutional Review Board approval was not required for this practice improvement project.

### Measures and data collection

After each session, a program satisfaction survey for the corresponding session was emailed through REDCap (Research Electronic Data Capture) to all attendees for that session, followed by three reminders to nonresponders. Demographic surveys, including questions about mentorship, professional network, and goal setting, were sent after the introductory session and at month 1. Program satisfaction surveys included items about whether the session's objectives were met. Surveys on leadership self-efficacy, motivation to lead, and well-being were sent after the introductory session, 1 month after program completion, and 3 months after program completion. The mean scores for different time points were reported, and individual scores for three outcomes were tracked throughout the program and follow-up period.

We measured leadership self-efficacy by using eight items developed by Kane and Baltes.^25^ We measured motivation to lead by using five items on affective-identity motivation developed by Chan and Drasgow.^26^ Both leadership self-efficacy and motivation to lead were measured using a 5-point Likert-type scale. After the introductory session, month 1, and 3, we used measured well-being using the Mayo Clinic Well-being Index, a well-validated, web-based self-assessment tool.^27^

### Statistical analyses

Descriptive statistical analyses were used to explore the outcome of program results.

## Results

Table 1 summarizes the demographics of participants. Of the 71 faculty members notified of the program, 41 (58%) attended the preprogram introductory session and committed to the program; out of 41 participants, 31 (76%) attended session 1; 25 (60%) attended session 2; 20 (49%) attended session 3; and 22 (54%) attended session 4. The response rates for the 1- and 3-month follow-up surveys were 21% and 17%, respectively.

**Table 1.** Participant Demographics

Variable	Introductory session, ***n*** (%) (***N*** = 41)
Current age range	
31–40 years	15 (36.6)
41–50 years	13 (31.7)
51–60 years	10 (24.4)
60 years or more	2 (4.9)
Did not wish to answer	1 (2.4)
Race and ethnicity	
White	26 (63.4)
Black	2 (4.9)
Asian	11 (26.8)
Middle Eastern or North African	1 (2.4)
Other	1 (2.4)
Did not wish to answer	3 (7.3)
Are you a caregiver in your personal life (please exclude your professional role)?	
Yes	15 (37.5)
No	25 (62.5)
Missing	1
Have you had any leadership experience?	
Yes	25 (61.0)
No	16 (39.0)
Have you ever participated in a leadership-focused program or meeting?	
Yes	23 (57.5)
No	17 (42.5)
Missing	1

Results from the initial survey showed increases in percentages for those who responded very good (7.3% to 13.3%) and excellent (19.5% to 40%) for having a robust professional network (introductory session to month 1) (Table 2). Those who responded favorably to having the courage to set and attain ambitious goals increased from 78.1% to 93.3%. Overall, 80% to 100% of participants favorably rated the session objectives (agreed or strongly agreed) (Table 3).

**Table 2.** Demographic Survey After the Introductory Session and at Month 1

	***n*** (%)	
Categories	Introductory session (***N*** = 41)	Month 1 (***n*** = 15)
The robustness of my professional network		
Excellent	8 (19.5)	6 (40.0)
Very good	3 (7.3)	2 (13.3)
Good	12 (29.3)	4 (26.7)
Fair	14 (34.1)	3 (20.0)
Poor	4 (9.8)	0 (0)
Relationship with at least 1 professional mentor		
Excellent	7 (17.1)	5 (33.3)
Very good	8 (19.5)	4 (26.7)
Good	10 (24.4)	5 (33.3)
Fair	9 (22.0)	1 (6.7)
Poor	7 (17.1)	0 (0)
I have the courage to set and attain ambitious professional goals		
Strongly agree	10 (24.4)	6 (40.0)
Agree	22 (53.7)	8 (53.3)
Neutral	6 (14.6)	1 (6.7)
Disagree	3 (7.3)	0 (0)

**Table 3.** Program Outcomes

Program satisfaction results for 4 sessions	
Session 1: Emotional intelligence (***n*** = 18)	Total ***n*** (%)
The session met the objective	
Identifying the 4 components of emotional intelligence	
Strongly agree	13 (72.2)
Agree	5 (27.8)
Understanding the emotional intelligence model and its impact on work performance	
Strongly agree	14 (77.8)
Agree	4 (22.2)
Able to increase your emotional intelligence	
Strongly agree	9 (50.0)
Agree	6 (33.3)
Neither agree nor disagree	3 (16.7)

**Session 2: Appreciative coaching (*n* = 13)**	
The session met the objective	
Clarifying the concept and the importance of workplace coaching	
Strongly agree	7 (53.8)
Agree	5 (38.5)
Strongly disagree	1 (7.7)
Understanding the critical coaching skills needed in practice	
Strongly agree	8 (61.5)
Agree	4 (30.8)
Strongly disagree	1 (7.7)
Able to provide ways in which we can use coaching skills with our colleagues	
Strongly agree	7 (53.8)
Agree	5 (38.5)
Strongly disagree	1 (7.7)

**Session 3: Resilience (*n* = 10)**	
The session met the objective	
Describing the lived experience of burnout among clinicians	
Strongly agree	8 (80.0)
Agree	2 (20.0)
Identifying contemporary factors that contribute to clinician burnout and its impact on professional practice	
Strongly agree	8 (80.0)
Agree	1 (10.0)
Neither agree nor disagree	1 (10.0)
Teaching how to develop skills that enhance both individual and team resilience	
Strongly agree	5 (50.0)
Agree	3 (30.0)
Neither agree nor disagree	1 (10.0)
Disagree	1 (10.0)
Reflecting on the effectiveness of mindfulness-based practices in managing daily stress	
Strongly agree	5 (50.0)
Agree	4 (40.0)
Neither agree nor disagree	1 (10.0)

**Session 4: Strategic career development (*n* = 12)**	
The session met the objective	
Describing the roadmap for personal and professional success	
Strongly agree	11 (91.7)
Agree	1 (8.3)
Teaching me how to identify and define my personal values, purpose, and vision	
Strongly agree	9 (75.0)
Agree	3 (25.0)
How to develop measurable	
Strongly agree	8 (66.7)
Agree	4 (33.3)
Showing me how to implement a personalized strategic plan to advance my personal and professional success	
Strongly agree	7 (58.3)
Agree	5 (41.7)

The program outcomes of leadership self-efficacy, motivation to lead, and well-being are described in Table 4. The total score for leadership self-efficacy was 45, motivation to lead was 25, and well-being was 7. Because of the substantially lower response rate after month 1 and 3, a column was added to compare the introductory session scores of the participants who completed month 1. The mean rating for leadership self-efficacy increased from 31.8 (32.9 for the same participants who rated after month 1) for the introductory session to 34.5 at month 1 and 35.1 at month 3. The total scores ranged from 20 to 45 (25–39 for the same participants who rated after month 1) to 29 to 42 after month 1 and 27 to 40 after month 3.

**Table 4.** Program Outcomes for Self-Efficacy, Motivation to Lead, and Well-Being

Category	Introductory session	Introductory session for those completing month 1	After month 1	After month 3
Leadership self-efficacy (possible score = 45)				
***n***	39	15	15	12
Mean (SD)	31.8 (5.6)	32.9 (4.7)	34.5 (4.2)	35.1 (4.1)
Range	20–45	25–39	29–42	27–40
Motivation to lead (possible score = 25)				
*n*	38	15	15	11
Mean (SD)	17.4 (3.6)	18.1 (3.8)	17.9 (3.9)	19.7 (2.9)
Range	8–24	12–24	11–23	16–25
Well-being (possible score = 7)				
*n*	40	15	15	12
Mean (SD)	2.4 (1.7)	3.1 (1.1)	2.9 (1.8)	4.1 (2.2)
Range	0–5	1–4	0–6	0–7

SD, standard deviation.

The scores in the lower range improved contributing to the overall increase in mean scores. The mean scores for motivation to lead did not increase at month 1 but increased by 2.3 points at month 3. The low-range scores improved from 8 as the lowest score at the introductory session to 16 at month 3. Mean scores for well-being increased from 2.4 to 2.9 at month 1 and to 4.1 at month 3. For well-being, the lower-range score remained the same but upper-range scores improved, contributing to the overall mean.

**FIG. 2.**
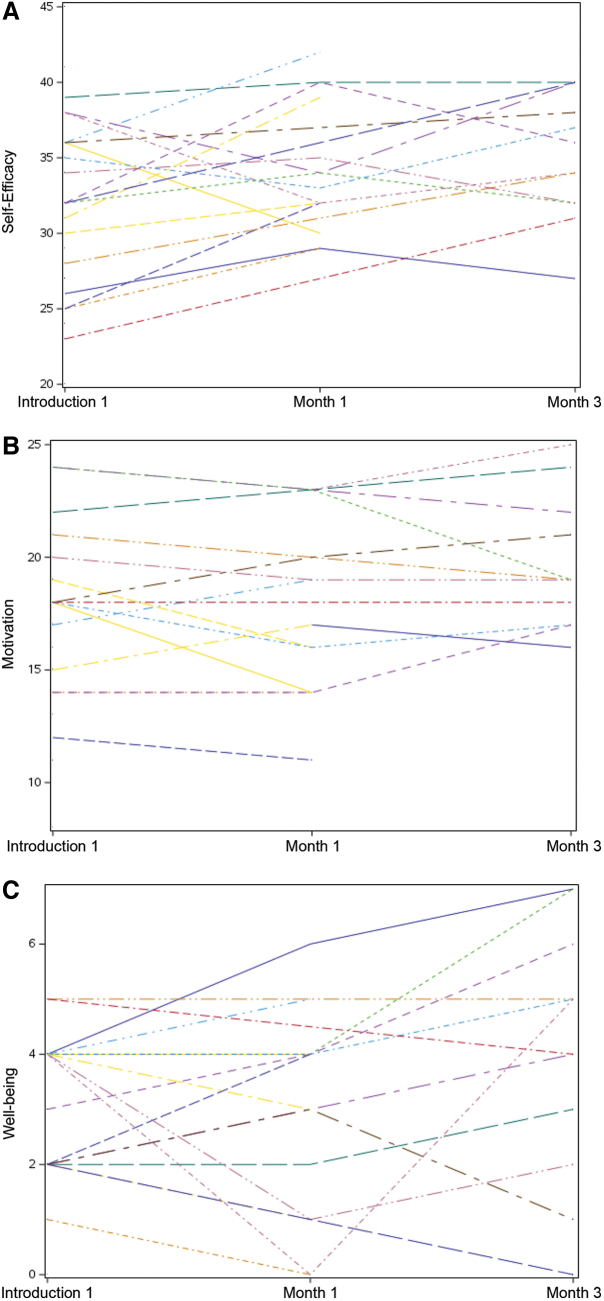
Individual Score Progression for 3 Program Outcomes (15 Participants). **(A)** Leadership self-efficacy (possible score = 45). **(B)** Motivation (possible score = 25). **(C)** Well-being (possible score = 7).

Figure 2 shows score progression for 3 program outcomes (self-efficacy, motivation to lead, and well-being) of the 15 participants who completed the month 1 survey. Some responded only to the introductory survey, and some responded only to the surveys after months 1 and 3. Except for two respondents, the ratings for leadership self-efficacy increased from the introductory session to month 3. Motivation to lead changed only slightly. Well-being scores fluctuated, likely because well-being is affected by daily needs and self-fulfillment. Of the 15 respondents, well-being increased for 10 respondents from the introductory session to months 1 or 3 or from month 1 to 3.

## Discussion

Among respondents, 80% to 100% felt that the unique session objectives were met. Many welcomed the opportunity to attend the sessions during the noon hour. For women, leadership development opportunities are rare, and prioritizing such activities are challenging with current workloads and other responsibilities. We were unable to assess application of the learning objectives (change in behavior), because the analysis would require more longitudinal observation.^28^ These are common issues in workplace education, and programs need to be closely aligned with ongoing support for women to be able to practice and implement what was learned in their own life context.^29^

Themes that were consistent throughout the program were importance of network, mentorship, and setting career development goals. The demographic survey at the introductory session and after month 1 clearly demonstrated that a greater percentage of respondents believed that they had a stronger professional network, better mentorship, and career development goals as a result of the program.

Leadership self-efficacy refers to “an individual's belief in his or her ability to succeed in the capacity of being a leader.”^25,30^ Self-efficacy for leadership is lower in women early in their academic careers than in men, and those women who had greater leadership self-efficacy more likely identified with leadership and withstood the negativity of stereotypes.^30,31^ In a literature review, leadership self-efficacy was suggested to have positive associations with potential, performance, and behavioral ratings of leaders.^32^ Leaders with greater general self-efficacy are known to be more effective and are inclined to expend more effort to fulfill their roles and to persevere longer when faced with difficulties.^33^

According to Bandura et al.,^34^ gender greatly affects self-efficacy because of inherent social expectations and social roles. Gender stereotypes influence leadership self-efficacy, and women leaders tend to underestimate their perceived leadership ability.^35,36^ Leadership self-efficacy, however, is not easy to change. Ng and Lucianetti^37^ suggested that organizational trust and perceived respect decrease anxiety and fear and build psychologically safe environments, which bring innovation, creativity, and growth to self-efficacy. We learned that networking with peers sharing common interests and challenges in a safe environment inspired others through reflective discussions and peer coaching and contributed to overall leadership self-efficacy.

Motivation to lead is defined as an individual's willingness to engage in leadership training activities and assume leadership roles.^26^ Our results showed that motivation to lead did not change much over the course of the program but increased after the program ended. We postulate that participants in the current program were motivated enough to participate despite their busy schedules, suggesting that they were motivated to begin with, as shown by the percentage of participants with leadership experience and with experience in leadership development programs. Studies on motivation to lead also show that motive to lead is shaped by relatively stable characteristics, such as personality and values, and in small part with past experiences that do not change by education programs.^26^

Our results for well-being fluctuated substantially over time, which is consistent with the understanding that well-being is flexible and constantly changing^38^ and is dependent upon many contextual factors that are often difficult to control in a work environment. Our results confirm that well-being at any point in time should be assessed cautiously and treated as a fluid state rather than as a stable trait. We postulated that the RISE 2.0 program would positively influence well-being based on the self-determination theory,^39–41^ which specifies that overall psychological well-being requires the satisfaction of three needs: autonomy, competence, and social relatedness.

The RISE 2.0 program sought to increase the level of participant autonomy and competence through peer coaching and to help participants develop emotional intelligence by being more self-aware and confident, which was empowering. Forming connections and a sense of belonging with a group of women leaders experiencing similar challenges and career aspirations contributed to the social relatedness factor.

### Limitations

One of the main challenges in conducting program evaluation studies is the lower response rates for after-program surveys. We aimed to gather enough responses to run rigorous statistical analyses of paired comparisons, which would have given us more definitive results of the program outcomes, but the low response rates negated this aim. Another of our challenges was the limited in-person interaction because of the coronavirus disease 2019 (COVID-19) pandemic. In-person interactions could have resulted in more sharing of experiences and challenges and in more peer support and learning.

## Conclusion

According to positive assessments and feedback received from participants, the RISE 2.0 program was promising in fostering the career and leadership progression of women in the medical field. Various topics such as emotional intelligence, resilience, appreciative coaching skills, strategic career development, and fostering social interactions garnered predominantly favorable reactions in achieving the outlined learning goals. However, the evaluations largely relied on subjective rather than objective measures, leaving the long-term outcomes such as career advancements and leadership performance yet to be observed. Nevertheless, building upon these initial accomplishments, we maintain confidence that the program will expand, uphold participant learning, and motivate leaders to persist in their paths toward successful and fulfilling careers.

## Abbreviations Used

COVID-19: coronavirus disease 2019
REDCap: Research Electronic Data Capture
RISE: Reflect, Inspire, Strengthen, and Empower
SD: standard deviation
